# Case Report: Synchronous gastric glomus tumor and gastrointestinal stromal tumor identified in the same gastric region

**DOI:** 10.3389/fmed.2026.1919970

**Published:** 2026-10-29

**Authors:** Chen Wang, Baosu Huang, Shunji Sun

**Affiliations:** 1 School of Clinical Medicine, Shandong Second Medical University, Weifang, Shandong, China; 2 Affiliated Hospital of Shandong Second Medical University, School of Clinical Medicine, Shandong Second Medical University, Weifang, Shandong, China

**Keywords:** diagnostic pitfall, gastric glomus tumor, gastrointestinal stromal tumor, immunohistochemistry, radiologic–endoscopic discordance

## Abstract

Gastric glomus tumors and gastrointestinal stromal tumors are uncommon gastric mesenchymal tumors that may present as subepithelial or gastric-wall lesions, making preoperative diagnosis challenging. We report a woman in her early 70 s who was admitted after a gastric mass was incidentally detected during routine physical examination. Magnetic resonance imaging and contrast-enhanced computed tomography showed a 3.8–4.3 cm heterogeneously enhanced gastric-wall or extraluminal mass adjacent to the lesser curvature, and a gastric stromal tumor was suspected. However, gastroscopy revealed only a 1.5 cm smooth intraluminal submucosal lesion at the gastric angle. The patient underwent combined laparoscopic and endoscopic surgery based on the available imaging and endoscopic findings. Endoscopic submucosal dissection was performed for the intraluminal lesion, and laparoscopic resection was performed for the larger gastric-wall mass. Histopathological and immunohistochemical examination of two separately submitted specimens confirmed a gastric glomus tumor and a low-risk gastric GIST identified in the same gastric region. Their precise spatial relationship could not be established from the separately submitted specimens. The postoperative course was uneventful. At telephone follow-up approximately 6 months after surgery, the patient reported no obvious gastrointestinal symptoms but had not completed the recommended objective surveillance. She was subsequently lost to follow-up; therefore, no endoscopic or radiological follow-up data were available. This case illustrates the value of correlating imaging, endoscopic, operative, and pathological findings in gastric mesenchymal tumors. Radiologic–endoscopic size discordance is nonspecific and does not by itself establish the presence of two lesions.

## Introduction

Gastric submucosal tumors comprise a heterogeneous group of lesions, among which gastrointestinal stromal tumors (GISTs) are the most common mesenchymal tumors of the gastrointestinal tract. Current guidelines emphasize that the diagnosis and management of GISTs should be based on tumor location, size, mitotic activity, histopathological features, immunohistochemical profile, and recurrence-risk assessment (1). In contrast, gastric glomus tumor (GGT) is an uncommon mesenchymal neoplasm derived from modified smooth muscle cells of the glomus body. Although most GGTs follow a benign clinical course, their nonspecific symptoms and submucosal growth pattern make preoperative diagnosis challenging (2).

Radiologically and endoscopically, GGTs may mimic GISTs and other gastric subepithelial lesions. Contrast-enhanced computed tomography may show marked or heterogeneous enhancement, reflecting the vascular nature of the tumor; however, these findings are not sufficiently specific for a definitive diagnosis (3). Therefore, histopathological examination and immunohistochemistry remain essential for differentiating GGT from GIST and other gastric mesenchymal tumors.

Here, we report a gastric glomus tumor and a low-risk gastric GIST that were identified in the same gastric region and resected separately. The case illustrates the challenges of correlating radiological and endoscopic findings with distinct postoperative pathological diagnoses.

## Case description

A woman in her early 70s was admitted to our department because a gastric mass had been incidentally detected during a routine physical examination 3 weeks earlier. She had no abdominal pain, abdominal distension, nausea, vomiting, hematemesis, melena, fever, or chills. Her medical history was notable for hypertension for more than 30 years, which was controlled with oral antihypertensive medication. Physical examination on admission revealed no obvious abdominal tenderness, rebound tenderness, or palpable abdominal mass.

## Diagnostic assessment

Preoperative laboratory tests showed no specific abnormalities related to the gastric lesion, and serum tumor markers, including carcinoembryonic antigen, carbohydrate antigen 19-9, carbohydrate antigen 125, and carbohydrate antigen 72-4, were within the normal ranges. EUS was not performed before resection. The treating team proceeded with operative management on the basis of CT/MRI and conventional gastroscopy, because the larger mass was considered resectable and surgical treatment was planned.

Preoperative magnetic resonance imaging showed a soft-tissue mass in the hepatogastric space, measuring approximately 3.8 × 3.0 cm. The lesion was adjacent to the lesser curvature of the stomach, with an unclear boundary from the gastric wall, and a gastric stromal tumor was considered likely. Contrast-enhanced computed tomography showed a heterogeneously enhanced soft-tissue mass in the hepatogastric space, measuring approximately 4.3 × 3.8 cm, with an indistinct boundary from the lesser curvature of the stomach (Figure 1A). The radiological impression also favored a gastric stromal tumor.

**Figure 1:**
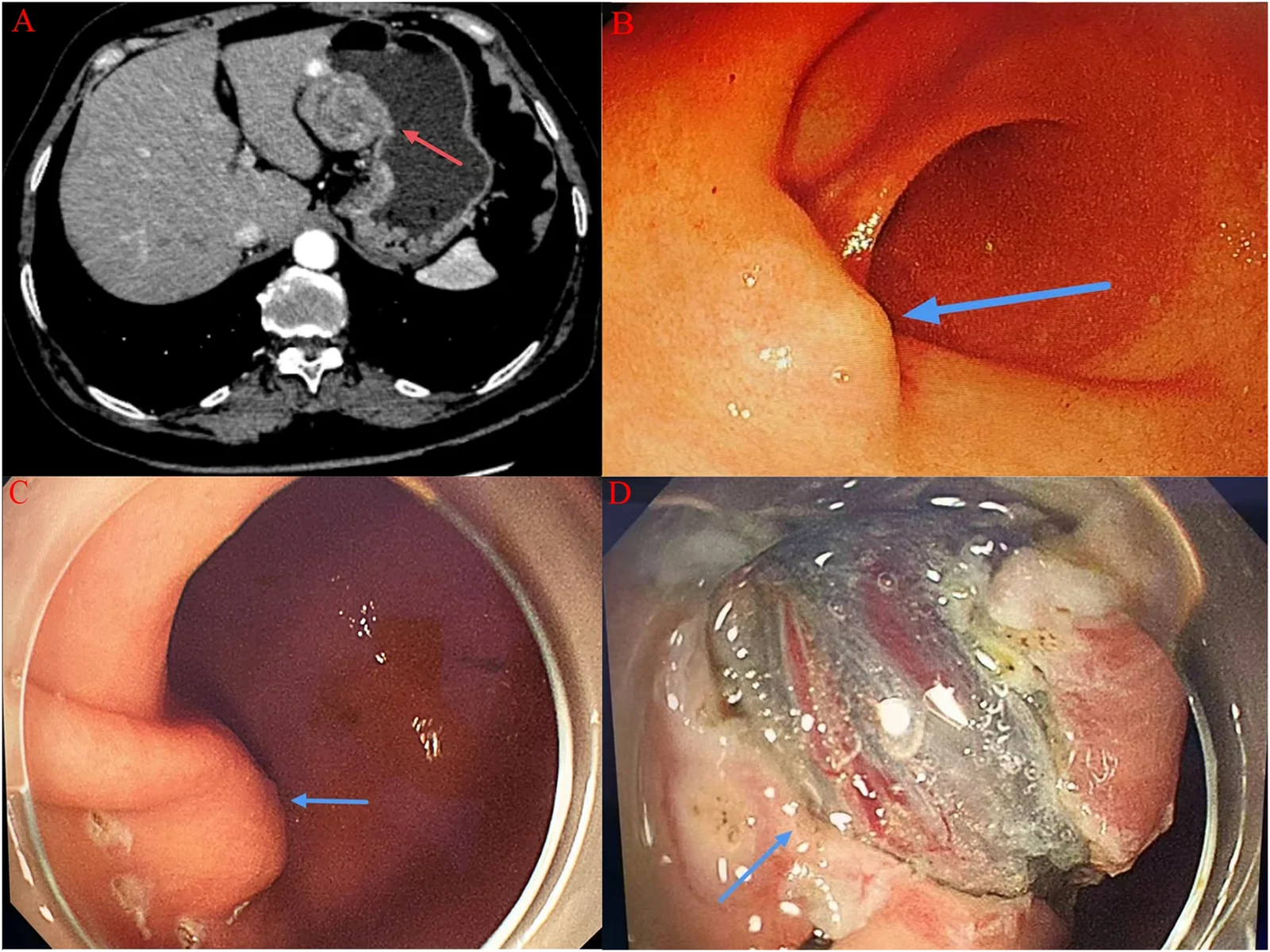
Radiologic and endoscopic findings. **(A)** Contrast-enhanced computed tomography showing a heterogeneously enhanced mass adjacent to the lesser curvature of the stomach. **(B)** Gastroscopic view showing a smooth submucosal lesion at the gastric angle. **(C)** Intraoperative endoscopic view of the submucosal lesion before resection. **(D)** Intraoperative endoscopic view after mucosal incision showing abundant surface vessels of the lesion.

Gastroscopy revealed an elevated lesion in the gastric angle region. A smooth, hard, fixed submucosal mass approximately 1.5 cm in diameter was observed on the anterior wall of the gastric angle, with intact overlying mucosa (Figure 1B). Helicobacter pylori testing was negative, and chronic non-atrophic gastritis was also noted. Colonoscopy revealed rectal polyps, which were later confirmed as hyperplastic polyps and were not considered related to the gastric lesions. The clinical course is summarized in Table 1.

**Table 1:** Timeline of the clinical course.

Time point	Clinical event	Key findings or management
3 weeks before admission	Incidental detection of gastric mass	A gastric mass was detected during routine physical examination.
Admission	Clinical evaluation	The patient had no abdominal pain, nausea, vomiting, hematemesis, melena, fever, or other gastrointestinal symptoms.
Preoperative laboratory assessment	Laboratory tests and tumor markers	No specific abnormalities related to the gastric lesion were identified.
Preoperative MRI	Cross-sectional imaging	MRI showed a 3.8 × 3.0 cm soft-tissue mass in the hepatogastric space adjacent to the lesser curvature, with an unclear boundary from the gastric wall. A gastric stromal tumor was suspected.
Preoperative CT	Contrast-enhanced imaging	CT showed a 4.3 × 3.8 cm heterogeneously enhanced mass adjacent to the lesser curvature, again suggesting a gastric stromal tumor.
Preoperative gastroscopy	Endoscopic assessment	Gastroscopy showed a smooth 1.5-cm submucosal lesion at the gastric angle, smaller than the mass measured on CT and MRI.
Same hospitalization	Therapeutic intervention	Combined laparoscopic and endoscopic surgery was performed, including endoscopic submucosal dissection of the intraluminal lesion and laparoscopic resection of the larger gastric-wall mass. Both lesions were considered macroscopically completely removed on intraoperative assessment.
Postoperative pathology	Final diagnosis	Two separately submitted specimens from the same gastric region were diagnosed as gastric glomus tumor and low-risk gastric gastrointestinal stromal tumor, respectively.
Postoperative course	Recovery	The patient recovered without major complications. The abdominal drain was removed on postoperative day 10, and she was discharged after a total 13-day hospitalization following gradual dietary advancement and inpatient observation.
Approximately 6 months after surgery	Outcome assessment	The patient reported good general condition and no obvious gastrointestinal symptoms but had not completed the recommended endoscopic or radiological reassessment. She was subsequently lost to follow-up, and no objective surveillance data were available.

## Therapeutic intervention

The combined approach was selected to permit endoscopic assessment and treatment of the intraluminal lesion together with laparoscopic evaluation and resection of the gastric-wall mass. During the same hospitalization, the patient underwent combined laparoscopic and endoscopic surgery under general anesthesia, including endoscopic submucosal dissection and laparoscopic gastric lesion resection. Intraoperatively, endoscopy showed the submucosal lesion before resection (Figure 1C). After mucosal incision, abundant surface vessels of the lesion were observed (Figure 1D). Laparoscopic exploration showed no visible peritoneal metastasis. A mass measuring approximately 4 × 5 × 3 cm was identified in the gastric angle region, with mild adhesion to the surrounding tissue. An ESD wound was visible beneath the gastric-wall mass during laparoscopy. This observation localized both treated sites to the same gastric-angle region, but did not establish their precise spatial or histological relationship. The larger mass was removed *en bloc* in a specimen bag, with no intraoperative tumor rupture or spillage. Both lesions were considered macroscopically completely removed on intraoperative assessment. The procedure was completed without blood transfusion, and the estimated blood loss was approximately 100 mL.

## Histopathological and immunohistochemical findings

Postoperative pathological examination revealed two distinct gastric lesions that had been removed and submitted as separate specimens; consequently, no shared tissue interface was available for microscopic evaluation. The first specimen, labeled as the gastric submucosal mass, measured approximately 1.2 × 1 × 1 cm. Hematoxylin and eosin staining showed uniform round tumor cells arranged around branching vascular channels, supporting the diagnosis of gastric glomus tumor (Figures 2A,B). Immunohistochemical staining showed CD117 negativity, SMA positivity (Figure 2C), vascular CD34 positivity (Figure 2D), CD56 negativity, chromogranin A negativity, focal synaptophysin positivity, partial h-caldesmon positivity, and a Ki-67 proliferation index of approximately 3% (Figure 2E). The available pathology report did not document a DOG1 result for the gastric glomus tumor.

**Figure 2:**
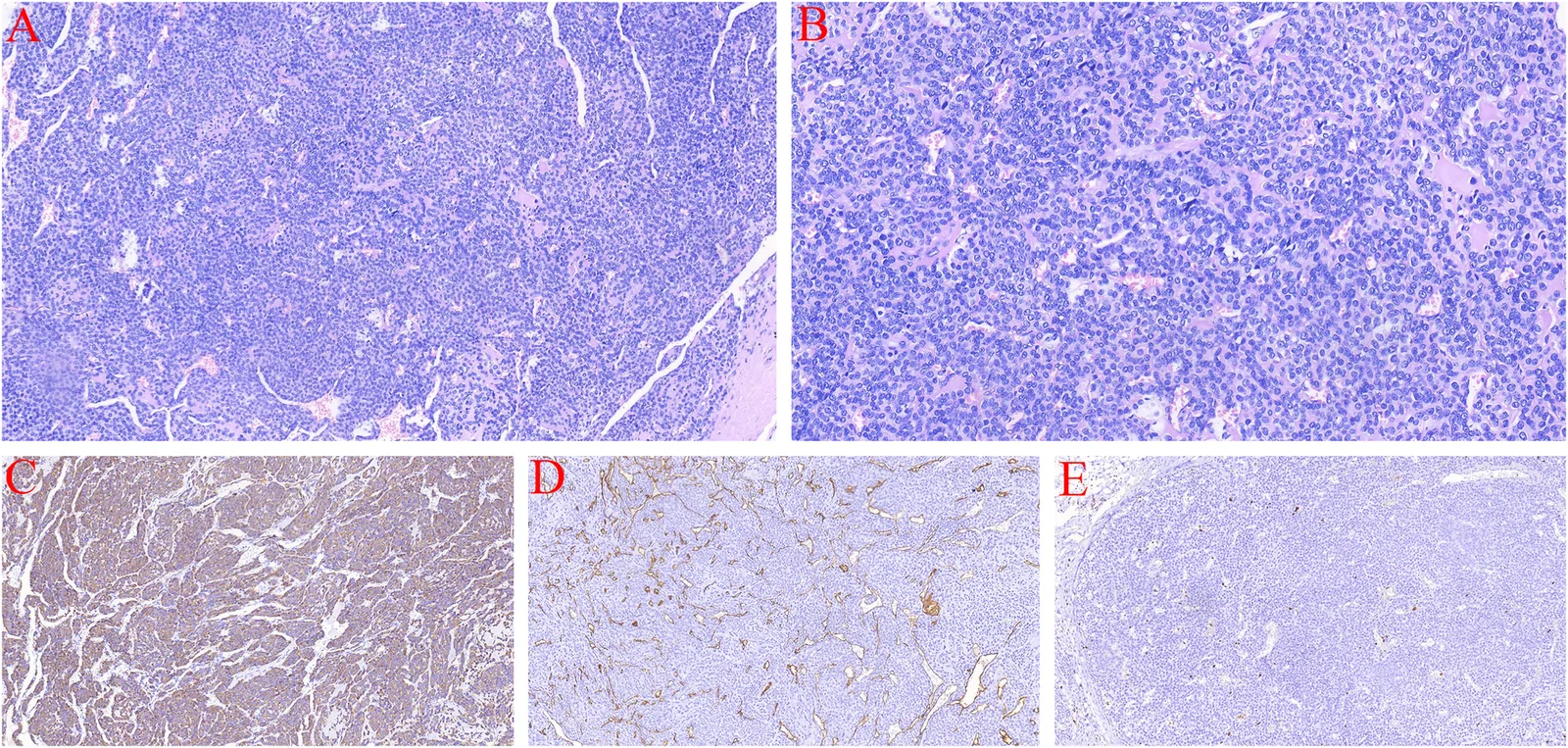
Histological and immunohistochemical findings of the gastric glomus tumor. **(A,B)** Hematoxylin and eosin staining showing uniform round tumor cells arranged around branching vascular channels (original magnification ×10 and ×20, respectively). **(C)** Smooth muscle actin showing cytoplasmic positivity in tumor cells. **(D)** CD34 highlighting vascular structures rather than tumor cells. **(E)** Ki-67 staining showing a proliferation index of approximately 3%.

The second specimen, labeled as the gastric mass, measured approximately 4 × 4 × 3.5 cm. Hematoxylin and eosin staining showed spindle cells arranged in intersecting fascicles, confirming a spindle-cell-type gastrointestinal stromal tumor (Figures 3A,B). The mitotic count was <2 mitoses per 5 mm^2^ in the most mitotically active area. Immunohistochemical staining showed DOG1 positivity (Figure 3C), CD117 positivity (Figure 3D), CD34 positivity (Figure 3E), S-100 negativity, desmin negativity, SMA negativity, vimentin positivity, and a Ki-67 proliferation index of approximately 5% (Figure 3F). Gross examination showed an intact tumor capsule. Microscopic resection-margin assessment was not performed; consequently, histologically complete excision and an R0 resection could not be confirmed. These findings supported the presence of synchronous gastric glomus tumor and low-risk gastric GIST rather than a single tumor type.

**Figure 3:**
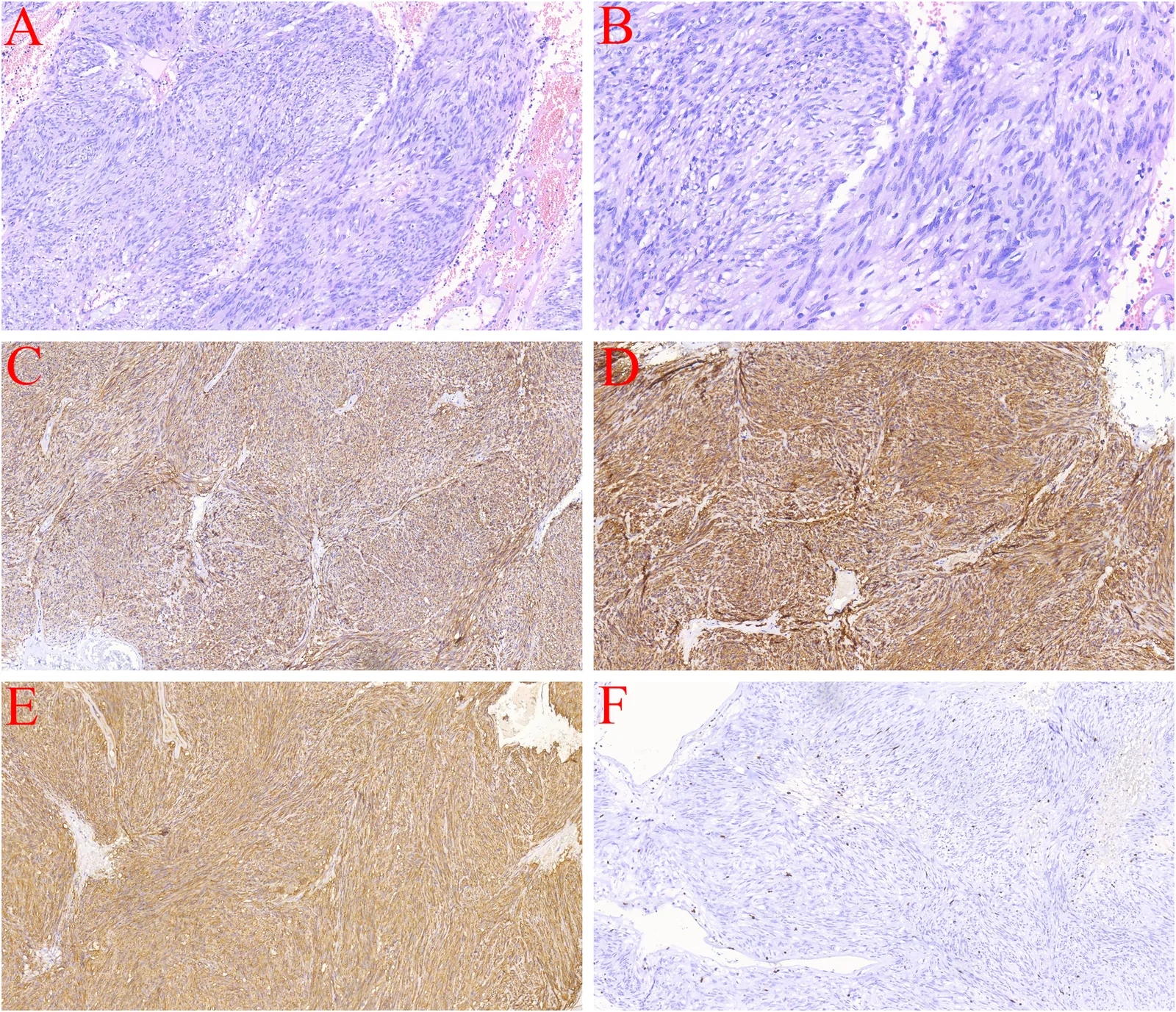
Histological and immunohistochemical findings of the gastric GIST. **(A,B)** Hematoxylin and eosin staining showing intersecting fascicles of spindle cells, consistent with a spindle-cell-type gastrointestinal stromal tumor (original magnification ×10 and ×20, respectively). The mitotic count was <2 mitoses per 5 mm^2^ in the most mitotically active area. **(C)** DOG1, **(D)** CD117, and **(E)** CD34 showing positive immunostaining in tumor cells. **(F)** Ki-67 staining showing a proliferation index of approximately 5%.

## Follow-up and outcomes

The patient recovered without major postoperative complications and was discharged after a 13-day hospitalization. The length of stay reflected postoperative management after the combined ESD and laparoscopic resection, including gradual resumption of oral intake and maintenance of an abdominal drain, which was removed on postoperative day 10; no adverse event prolonged the hospitalization. At discharge, regular outpatient follow-up and objective endoscopic or radiological surveillance were recommended. At telephone follow-up approximately 6 months after surgery, the patient reported good general condition and no obvious gastrointestinal symptoms but had not attended the recommended objective reassessment. She was subsequently lost to follow-up; consequently, no objective postoperative endoscopic or radiological data were available.

## Discussion

Radiologic–endoscopic size discordance is nonspecific. A single predominantly exophytic tumor may be visible on cross-sectional imaging while producing little or no abnormality on conventional gastroscopy (4). In the present case, the two diagnoses were established by histopathological and immunohistochemical examination of separately resected specimens. The discrepancy in size alone did not establish two lesions or their precise spatial relationship. The clinical lesson is the importance of correlating findings across modalities and obtaining an appropriate pathological assessment of each resected specimen.

This case is also relevant to multidisciplinary decision-making. In routine practice, gastric subepithelial lesions are often evaluated by different specialists, and radiologic, endoscopic, surgical, and pathological information may be interpreted separately. When these findings are not concordant, important diagnostic clues may be missed. In the present case, the endoscopic lesion was substantially smaller than the mass suggested by cross-sectional imaging, and this discrepancy became more meaningful only after the intraoperative and pathological findings were reviewed together. Therefore, the case supports a practical approach in which discordant findings should be actively reconciled before and during treatment. For suspected gastric mesenchymal tumors, clinicians should review whether the endoscopic appearance, imaging size, lesion location, and operative findings can all be explained by a single lesion. If not, careful intraoperative localization, separate specimen submission, and targeted pathological assessment are particularly important. Multimodal correlation complements preoperative characterization with EUS and, when appropriate, tissue acquisition; this case does not establish the adequacy of omitting these investigations.

Preoperative diagnosis of gastric glomus tumor remains difficult. GGTs are rare and often present as subepithelial or submucosal lesions with nonspecific clinical manifestations. Some patients present with epigastric discomfort or gastrointestinal bleeding, whereas others are asymptomatic and diagnosed incidentally, as in the present case (2, 5). Contrast-enhanced CT may show marked or heterogeneous enhancement because of the vascular nature of the tumor, but this feature is not specific enough to reliably distinguish GGT from GIST or other gastric subepithelial lesions (3). In this patient, the larger lesion was located in the hepatogastric space and showed heterogeneous enhancement with an indistinct boundary from the lesser curvature of the stomach, which reasonably led to a preoperative impression of gastric stromal tumor.

EUS is useful for characterizing gastric subepithelial lesions, including their layer of origin, internal echogenicity, margins, and relationship with the gastric wall (5, 6). In this case, its absence limited preoperative anatomical characterization. EUS might have helped determine whether the imaging and endoscopic findings represented different components of a single lesion or separate lesions, and could have informed the choice of resection technique. The decision to proceed without EUS in this individual case should not be interpreted as evidence that EUS was unnecessary.

The final diagnosis in this case depended on histopathology and immunohistochemistry. Gastric glomus tumors show perivascular smooth-muscle differentiation and commonly express SMA and h-caldesmon. CD117 is usually negative, whereas focal DOG1 expression has been reported; diagnosis therefore requires correlation of morphology with the overall immunohistochemical profile (2, 7, 8). In this patient, the smaller gastric submucosal lesion showed uniform round cells arranged around branching vascular channels (Figures 2A,B). Immunohistochemistry showed SMA positivity (Figure 2C), partial h-caldesmon positivity, CD117 negativity, and a Ki-67 proliferation index of approximately 3% (Figure 2E), supporting the diagnosis of GGT. CD34 staining highlighted vascular structures rather than tumor cells (Figure 2D).

In contrast, the larger gastric wall mass was confirmed as a spindle-cell-type GIST and showed a typical immunophenotype (Figures 3A–F), with DOG1, CD117, and CD34 positivity and negativity for S-100, desmin, and SMA. The tumor measured approximately 4 × 4 × 3.5 cm. The mitotic count was <2 mitoses per 5 mm^2^ in the most mitotically active area. The original pathological report classified the gastric GIST as low risk. The intact gross capsule and absence of intraoperative rupture or spillage further informed recurrence-risk assessment. This clear histological and immunohistochemical contrast between the separately submitted specimens supports two distinct pathological entities rather than a single tumor type.

Although most gastric glomus tumors are benign, rare malignant or metastatic cases have been reported. Tumor size, deep location, atypical mitotic figures, nuclear atypia, and increased mitotic activity have been used to evaluate malignant potential in glomus tumors (9). In the present case, the GGT was small, with a low Ki-67 index, and no malignant features were described in the available pathological report. Nevertheless, follow-up remains appropriate because of the rarity of GGT and the coexistence of a low-risk GIST.

Deng et al. reported a patient with a gastric glomus tumor and a concurrent duodenal GIST in their series of 15 gastric glomus tumors (8). This report documents coexistence at different gastrointestinal sites and provides related clinical context, but does not establish the novelty of the present combination. In the present case, a gastric glomus tumor and a low-risk gastric GIST were identified in the same gastric region and resected separately, although their precise spatial relationship could not be established. The case illustrates the importance of correlating imaging, endoscopic, operative, and pathological findings when assessing gastric mesenchymal tumors.

The treatment strategy in this case was also clinically meaningful. Combined laparoscopic and endoscopic surgery allows endoscopic visualization of the intraluminal lesion while enabling laparoscopic management of the gastric wall or extraluminal component. Laparoscopic and endoscopic cooperative surgery and related combined approaches have been reported as useful minimally invasive strategies for selected gastric submucosal tumors, especially when precise localization and function-preserving resection are needed (10, 11). Although the operative context differs, literature on incidental gastric GISTs encountered during bariatric surgery similarly indicates that tumor size and location may alter the planned operation and supports individualized oncologic decision-making (12–14). In this patient, the combined approach enabled treatment of the intraluminal lesion and gastric-wall mass during the same operation. Its long-term oncological effectiveness could not be evaluated because microscopic margin status and objective postoperative surveillance were unavailable.

This case has several limitations. First, because EUS was not performed, preoperative characterization of the layer of origin and spatial relationship between the two lesions was limited. Second, follow-up was limited to a single telephone contact approximately 6 months after surgery. Current risk-adapted guidance notes that, if routine follow-up is selected for low-risk GIST, abdominal CT, MRI, or ultrasonography every 6–12 months for 5 years, followed by annual surveillance up to 10 years, may be considered (15). However, the patient had not undergone the recommended objective endoscopic or radiological surveillance and was subsequently lost to follow-up, precluding an objective assessment of recurrence. Third, because the tumors were removed and submitted as separate specimens, no shared histological interface was available. Neither intervening normal gastric tissue nor microscopic tissue bridging could therefore be assessed. The findings support two separately resected tumors identified in the same gastric region, but their precise spatial relationship and classification as a collision tumor could not be established. Fourth, KIT and PDGFRA mutational analysis was not performed. Although molecular profiling could have provided additional biological information, it was not required to guide immediate postoperative therapy for this low-risk gastric GIST without tumor rupture; its absence nevertheless limits molecular characterization. Finally, microscopic resection-margin status was not assessed, preventing confirmation of an R0 resection. The absence of objective postoperative surveillance also limits assessment of residual or recurrent disease.

## Conclusion

This case documents a gastric glomus tumor and a low-risk gastric GIST identified in the same gastric region and resected separately. Their precise spatial relationship could not be established. Radiologic–endoscopic size discordance is nonspecific and warrants careful correlation of imaging, endoscopic, and operative findings. Histopathological and immunohistochemical examination of each specimen was essential for establishing the two diagnoses.

## Patient perspective

A patient-authored account of her experience was unavailable because she was subsequently lost to follow-up. During telephone contact approximately 6 months after surgery, she reported good general condition and no obvious gastrointestinal symptoms but had not completed the recommended objective surveillance.

## Reporting guidelines

This case report was prepared in accordance with the CARE reporting guideline. The completed CARE checklist is provided as Supplementary Material.

## Data Availability

The original contributions presented in the study are included in the article/Supplementary Material, further inquiries can be directed to the corresponding author.

## References

[1] Casali PG Blay JY Abecassis N Bajpai J Bauer S Biagini R. Gastrointestinal stromal tumours: ESMO-EURACAN-GENTURIS clinical practice guidelines for diagnosis, treatment and follow-up. Ann Oncol. (2022) 33(1):20–33. 10.1016/j.annonc.2021.09.005 34560242

[2] Lin J Shen J Yue H Li Q Cheng Y Zhou M. Gastric glomus tumor: a clinicopathologic and immunohistochemical study of 21 cases. Biomed Res Int. (2020) 2020:5637893. 10.1155/2020/5637893 32337257 PMC7157787

[3] Xing JJ Huang WP Wang F Chai YR Gao JB. Computed tomography features and clinicopathological characteristics of gastric glomus tumor. BMC Gastroenterol. (2022) 22(1):174. 10.1186/s12876-022-02241-w 35397495 PMC8994361

[4] Aoe Y Sugihara T Ogawa A Nagahara R Miyoshi K Matono T. Natural history of exophytic type gastrointestinal stromal tumor: a case report. Yonago Acta Med. (2019) 62(1):166–8. 10.33160/yam.2019.03.024 30962761 PMC6437418

[5] Bai B Mao CS Li Z Kuang SL. Endoscopic ultrasonography diagnosis of gastric glomus tumors. World J Clin Cases. (2021) 9(33):10126–33. 10.12998/wjcc.v9.i33.10126 34904082 PMC8638066

[6] Sharzehi K Sethi A Savides T. AGA clinical practice update on management of subepithelial lesions encountered during routine endoscopy: expert review. Clin Gastroenterol Hepatol. (2022) 20(11):2435–43.e4. 10.1016/j.cgh.2022.05.054 35842117

[7] Miettinen M Paal E Lasota J Sobin LH. Gastrointestinal glomus tumors: a clinicopathologic, immunohistochemical, and molecular genetic study of 32 cases. Am J Surg Pathol. (2002) 26(3):301–11. 10.1097/00000478-200203000-00003 11859201

[8] Deng M Luo R Huang J Luo Y Song Q Liang H. Clinicopathologic features of gastric glomus tumor: a report of 15 cases and literature review. Pathol Oncol Res. (2023) 28:1610824. 10.3389/pore.2022.1610824 36699621 PMC9868133

[9] Folpe AL Fanburg-Smith JC Miettinen M Weiss SW. Atypical and malignant glomus tumors: analysis of 52 cases, with a proposal for the reclassification of glomus tumors. Am J Surg Pathol. (2001) 25(1):1–12. 10.1097/00000478-200101000-00001 11145243

[10] Hiki N Nunobe S. Laparoscopic endoscopic cooperative surgery (LECS) for the gastrointestinal tract: updated indications. Ann Gastroenterol Surg. (2019) 3(3):239–46. 10.1002/ags3.12238 31131352 PMC6524076

[11] Hanayama H Katagata M Sato T Nakano H Matsumoto T Tada T. Clinical outcomes of laparoscopic and endoscopic cooperative surgery for gastric gastrointestinal stromal tumor. Fukushima J Med Sci. (2022) 68(3):169–74. 10.5387/fms.2021-25 36543179 PMC9840883

[12] Abokhozima A Zidan MH Altabbaa H Abo Elmagd A Alokl M Fathy F. Can incidental gastric GISTs during bariatric surgeries change the primary plan of surgery? A single team experience and a systematic review of literature. Obes Surg. (2024) 34(6):2186–97. 10.1007/s11695-024-07224-2 38684584 PMC11127811

[13] El-Masry H Abouegylah M Abokhozima A. Critical aspects into surgical management, classification, and therapeutic guidelines for incidental GISTs during bariatric surgeries. Obes Surg. (2025) 35(7):2762–4. 10.1007/s11695-025-07919-0 40389744 PMC12271279

[14] Zidan MH El-Masry H Elmagd AA Alokl M Abokhozima A. Neglecting evidence and systematic review protocols while oversimplifying management: a critical appraisal of incidental GISTs in patients undergoing bariatric surgery. Obes Surg. (2025) 35(3):1199–200. 10.1007/s11695-025-07759-y 39985713

[15] Judson I Jones RL Wong NACS Dileo P Bulusu R Smith M. Gastrointestinal stromal tumour (GIST): British Sarcoma Group clinical practice guidelines. Br J Cancer. (2025) 132:1–10. 10.1038/s41416-024-02672-0 38840030 PMC11723931

