# Supplementary material for: Case Report: Synchronous gastric glomus tumor and gastrointestinal stromal tumor identified in the same gastric region
Source: Front Med (Lausanne). 2026 Oct 29;13:1919970. doi: 10.3389/fmed.2026.1919970 (PMC13758385; doi:10.3389/fmed.2026.1919970)
Supplement: Supplementary file 1 [file Table1.docx]

**CARE Checklist (2013) of information to include when writing a case report**

| **Topic** | **Item** | **Checklist item description** | **Reported in section** |
| --- | --- | --- | --- |
| **Title** | **1** | The words “case report” should be in the title along with what is of greatest interest in this case | Title |
| **Key Words** | **2** | The key elements of this case in 2 to 5 key words | Keywords |
| **Abstract** | **3a** | Introduction—What is unique about this case? What does it add to the medical literature? | Abstract |
|  | **3b** | The main symptoms of the patient and the important clinical findings | Abstract |
|  | **3c** | The main diagnoses, therapeutics interventions, and outcomes | Abstract |
|  | **3d** | Conclusion—What are the main “take-away” lessons from this case? | Abstract |
| **Introduction** | **4** | Brief background summary of this case referencing the relevant medical literature | Introduction |
| **Patient Information** | **5a** | Demographic information (such as age, gender, ethnicity, occupation) | Case description |
|  | **5b** | Main symptoms of the patient (his or her chief complaints) | Case description |
|  | **5c** | Medical, family, and psychosocial history including co-morbidities, and relevant genetic information | Case description |
|  | **5d** | Relevant past interventions and their outcomes | Not applicable |
| **Clinical Findings** | **6** | Describe the relevant physical examination (PE) findings | Case description |
| **Timeline** | **7** | Depict important milestones related to your diagnoses and interventions (table or figure) | Table 1 (Clinical timeline) |
| **Diagnostic Assessment** | **8a** | Diagnostic methods (such as PE, laboratory testing, imaging, questionnaires) | Diagnostic assessment |
|  | **8b** | Diagnostic challenges (such as financial, language, or cultural) | Diagnostic assessment; Discussion |
|  | **8c** | Diagnostic reasoning including other diagnoses considered | Diagnostic assessment; Histopathological and immunohistochemical findings; Discussion |
|  | **8d** | Prognostic characteristics (such as staging in oncology) where applicable | Histopathological and immunohistochemical findings; Discussion |
| **Therapeutic Intervention** | **9a** | Types of intervention (such as pharmacologic, surgical, preventive, self-care) | Therapeutic intervention |
|  | **9b** | Administration of intervention (such as dosage, strength, duration) | Therapeutic intervention |
|  | **9c** | Changes in intervention (with rationale) | Not applicable |
| **Follow-up and Outcomes** | **10a** | Clinician-assessed outcomes and when appropriate patient-assessed outcomes | Follow-up and outcomes; Patient perspective |
|  | **10b** | Important follow-up test results | Follow-up and outcomes (objective surveillance not performed) |
|  | **10c** | Intervention adherence and tolerability (How was this assessed?) | Follow-up and outcomes |
|  | **10d** | Adverse and unanticipated events | Therapeutic intervention; Follow-up and outcomes |
| **Discussion** | **11a** | Discussion of the strengths and limitations in the management of this case | Discussion |
|  | **11b** | Discussion of the relevant medical literature | Introduction; Discussion |
|  | **11c** | The rationale for conclusions (including assessment of possible causes) | Discussion; Conclusion |
|  | **11d** | The main “take-away” lessons of this case report | Discussion; Conclusion |
| **Patient Perspective** | **12** | Did the patient share his or her perspective or experience? (Include when appropriate) | Patient perspective |
| **Informed Consent** | **13** | Did the patient give informed consent? Please provide if requested | Ethics statement; Consent for publication (written consent obtained) |
